# Hybrid sequencing reveals incompleteness of the H37Rv reference genome and highlights lineage-specific genomic divergence in Mycobacterium tuberculosis

**DOI:** 10.1099/mgen.0.001688

**Published:** 2026-06-01

**Authors:** Haiting Chen, Xiangchen Li, Ruiqi Chen, Mingwu Zhang, Yelei Zhu, Kunyang Wu, Yewei Lu, Xiaomeng Wang, Yang Che, Zhengwei Liu

**Affiliations:** 1 School of Public Health, Hangzhou Medical College, Hangzhou, Zhejiang 310053, PR China; 2 Key Laboratory of Precision Medicine in Diagnosis and Monitoring Research of Zhejiang Province, Hangzhou 310016, PR China; 3 Department of Tuberculosis Control and Prevention, Zhejiang Provincial Center for Disease Control and Prevention, Hangzhou, Zhejiang 310051, PR China; 4 Institute of Tuberculosis Prevention and Control, Ningbo Municipal Center for Disease Control and Prevention, Ningbo, Zhejiang 315000, PR China

**Keywords:** genomic variation, hybrid sequencing, molecular epidemiology, *Mycobacterium tuberculosis*, reference genome

## Abstract

**Background.** The *Mycobacterium tuberculosis* H37Rv reference genome (Lineage 4), sequenced in 1998, may be incomplete and inaccurate due to earlier technology. Moreover, its use is problematic in regions like East Asia where the dominant Beijing lineage (Lineage 2) is prevalent, as it may introduce biases due to lineage-specific genetic differences.

**Methods.** We performed hybrid sequencing to generate high-quality assemblies of a clinically isolated Beijing family strain (A2018772) and a Chinese laboratory-adapted H37Rv strain (H37Rv_CN). Comparative genomic analysis was conducted against the standard H37Rv reference. The impact of reference genome choice on transmission clustering was evaluated using a dataset of 3,359 *M. tuberculosis* Lineage 2 strains from China.

**Results.** Comprehensive genomic analysis revealed substantial differences between both studied strains and H37Rv_ref. The Chinese laboratory-adapted H37Rv_CN strain differed by 145 SNPs, 49 InDels and 14 structural variants from the reference genome. The Beijing family clinical isolated strain A2018772 showed even greater divergence with 2,277 SNPs, 373 InDels and 219 structural variants compared to H37Rv_ref, with particular enrichment in PE/PPE genes and metabolic pathways. Among these variations, a 9.2 kb deletion was prevalent in 89.7% of 78 publicly available Lineage 2 genomes. When applied to transmission analysis of 3,359 Chinese Lineage 2 strains, the A2018772 as a reference genome demonstrated superior performance with higher mapping rates (99.52% vs. 98.72%) and coverage (99.73% vs. 98.50%) compared to H37Rv_ref. This improved resolution resulted in fewer transmission clusters (273 vs. 285) at the 5-SNP threshold, indicating that H37Rv_ref consistently overestimates transmission events in Lineage 2-dominated populations.

**Conclusion.** Our study demonstrates the incompleteness of the H37Rv reference genome and also finds that using a lineage-specific reference genome could significantly improve transmission inference accuracy for Lineage 2 strains, highlighting the need for region-specific genomic references to enhance tuberculosis control strategies in high-burden areas.

**Impact Statement**

Accurate and representative reference genomes are crucial for robust genomic epidemiology of *Mycobacterium tuberculosis* (MTB). The long-standing H37Rv reference, derived from Lineage 4 and sequenced using earlier technology, is probably lacking in completeness and may poorly reflect the genetic features of the predominant Lineage 2 (Beijing family) strains circulating in East Asia. In this study, we generated high-quality hybrid assemblies of a clinical Beijing strain and a Chinese laboratory-adapted H37Rv, revealing extensive lineage-specific sequence and structural variations. Using a comprehensive dataset of 3,359 Lineage 2 isolates from China, we demonstrated that employing a lineage-appropriate reference genome significantly improved mapping accuracy, genome coverage and transmission clustering resolution. Our findings highlight that reliance on the outdated H37Rv reference introduces systematic biases in genomic analyses and overestimates transmission events. This work emphasizes the importance of adopting updated, lineage- and region-specific reference genomes to improve the accuracy of MTB genomic epidemiology, thereby enhancing tuberculosis surveillance and control efforts in high-burden regions.

## Data Availability

Data Collection: Whole-genome sequencing data for the strains used in this study have been deposited in the EMBL Sequence Read Archive via project number PRJEB109064. H37Rv_CN (accession number: GCA_982191695E) and A2018772 (accession number: GCA_982191705) are publicly accessible at https://www.ebi.ac.uk/. FASTA files can be found in the following: (https://doi.org/10.6084/m9.figshare.31236682)[1].

## Introduction

Tuberculosis (TB), caused by the *Mycobacterium tuberculosis* complex (MTBC), is set to reclaim its status as the leading cause of death from a single infectious agent, after being surpassed by Coronavirus diease 2019. According to the World Health Organization (WHO), ~10.8 million people suffered from TB globally in 2023 [2]. Urgent action is required to achieve the 2030 global TB elimination target.

The MTBC genome (4.4 Mb, 60 mol% GC content) is highly conserved, with limited sequence diversity among strains [3]. MTBC strains adapted to humans are classified into ten phylogenetic lineages (L1 to L10), comprising ‘ancestral’ (L1, L5–L10) and ‘modern’ (Lineage 2, L3 and L4) lineages, distinguished by the presence or absence of the TbD1 genomic region [4,5]. Among these, L1 to L4 account for most global TB cases, representing 97.38% of the total. Specifically, L1 accounts for 9.73%, Lineage 2 for 26.73%, L3 for 10.55% and L4 for 50.38%. Notably, Lineage 2 shows a significantly higher prevalence in certain regions, such as Asia (54.4%) and Oceania (70.5%) [6]. Clinical and epidemiological studies indicate that MTBC lineages vary in virulence, transmissibility and disease outcomes, with distinct differences between ancestral and modern lineages [7]. These differences likely stem from long-term co-evolution with human hosts and geographic genetic isolation. Regionally, MTBC lineages show distinct distributions, with Lineage 2 (Beijing family) endemic in East Asia (China, South Korea and eastern Russia) and L4 predominant in Europe, North and South America and Africa [8,10]. Tracking lineage distribution is critical for predicting resistance patterns, improving diagnostics and optimizing treatment to strengthen TB control [11].

In China, Lineage 2 (Beijing family) predominates and plays a significant role in TB epidemiology, exhibiting high virulence and elevated mutation rates [12,13]. MTBC members evolved from environmental organisms into obligate pathogens through genomic streamlining and acquisition of novel genetic elements [14]. Previous studies have identified greater genetic diversity in Lineage 2 strains compared to L4, yet the extent of gene content variation between these lineages remains underexplored [15]. This gap limits our understanding of how these differences impact comprehensive genomic analyses, particularly for Lineage 2 strains.

Whole-genome sequencing (WGS) is central to MTBC strain analysis, typically aligning short reads to a reference genome like H37Rv or a reconstructed ancestral genome [16,18]. SNPs derived from this process are used to assess drug resistance, identify strain subtypes and study transmission dynamics [16]. However, the H37Rv reference genome, sequenced in 1998 using Sanger sequencing, may have limitations due to first-generation sequencing technology. As a Lineage 4 strain, H37Rv may not fully capture the genomic diversity of other lineages, particularly Lineage 2, potentially missing lineage-specific genes or regions critical for accurate analysis. Relying solely on H37Rv could skew genetic diversity assessments and hinder transmission studies. The suitability of lineage-specific reference genomes for WGS in regions where non-L4 lineages predominate requires further investigation.

To address these challenges, we evaluated gene content diversity in Lineage 2 strains using a hybrid sequencing approach combining short-read and long-read technologies to achieve high-resolution genomic assemblies. We compared a representative Lineage 2 Beijing family strain and the laboratory-standard H37Rv strain against the H37Rv reference genome (H37Rv_ref). Our study aimed to identify genomic particularities in Lineage 2 strains relative to L4-based H37Rv, assess discrepancies between the Chinese laboratory-standard H37Rv and the internationally sequenced H37Rv_ref and evaluate the impact of reference genome selection on transmission analysis. Using SNP distance matrices, we constructed transmission clusters with both Lineage 2 and L4 reference genomes to compare their influence on transmission dynamics. These findings could guide the development of region-specific reference genomes and improve global TB surveillance and control.

### Methods

### Bacterial strains

Laboratory stock of *M. tuberculosis* H37Rv (H37Rv_CN) was obtained from the Tuberculosis Reference Laboratory, Chinese Center for Disease Control and Prevention. The Beijing family strain A2018772, isolated from Linhai Hospital in Taizhou City, Zhejiang Province, China, was fully drug-susceptible. The H37Rv strain sequences (H37Rv_ref, NC_000962.3), sourced from the BAC library used for the 1998 reference genome sequence, were obtained from Institut Pasteur, France. WGS data for 3,359 Lineage 2 strains were obtained from the China National Drug-Resistant Tuberculosis Surveillance Program, collected between 2015 and 2020 across diverse geographic regions (https://ngdc.cncb.ac.cn/gsa/browse/CRA017099).

### Bacterial strain culture and DNA extraction

H37Rv_CN and A2018772 were re-cultured in Löwenstein–Jensen (LJ) solid medium. MTB colonies scraped from the LJ medium were resuspended in 500 µl Tris-EDTA buffer in an Eppendorf tube and inactivated at 80°C for 30 min. Inactivated pellets were frozen at −80°C overnight, thawed and treated with 50 µl lysozyme (100 mg ml^−1^) at 37°C for 24 h with gentle stirring to prevent DNA shearing. SDS (70 µl, 10% w/v) and proteinase K (15 µl, 20 mg ml^−1^) were added, and samples were incubated at 60°C for 1 h. CTAB/NaCl buffer (CTAB 40 mM, NaCl 0.6 M final concentration) was added, followed by incubation at 60°C for 15 min and centrifugation at 12,000 r.p.m. for 5 min. Supernatants were mixed with 750 µl chloroform/isoamyl alcohol (24:1 v/v), inverted gently and centrifuged at 12,000 r.p.m. for 5 min. DNA was precipitated with 700 µl pre-chilled 100% isopropyl alcohol, pelleted at 12,000 r.p.m. for 2 min at 4°C, dried at room temperature and resuspended in 50 µl nuclease-free water.

### Genomic DNA quality control

Genomic DNA (gDNA) quality for H37Rv_CN and A2018772 was assessed using a NanoDrop One™ spectrophotometer (Thermo Fisher Scientific, Waltham, MA, USA) for concentration and purity (A260/A280 ratio: 1.8–2.0; A260/A230 ratio: 1.5–2.2). The 4200 TapeStation system (Agilent Technologies, Santa Clara, CA, USA) was used to evaluate fragment size distribution and DNA integrity number (DIN). All samples showed a single high molecular weight peak, DIN >5 and sufficient concentration for downstream applications.

### Short-read genome sequencing and analysis

gDNA was fragmented to ~200–300 bp using a QIAamp DNA Mini Kit (Qiagen, Hilden, Germany). Libraries were prepared using Illumina TruSeq adapters and kits and sequenced on an Illumina NovaSeq 6000 platform (Illumina Inc., San Diego, CA, USA) with 150 bp paired-end reads. Raw reads were processed with fastp, quality-checked with FastQC and aligned to the H37Rv reference genome using BWA-MEM (v0.7.17). Variants were called using FreeBayes, and phylogenetic analysis was performed with Snippy.

### Long-read genome sequencing and analysis

Long-read sequencing was employed to resolve complex genomic regions, such as repetitive sequences, that are challenging for short-read sequencing alone. Libraries with a 10 kb insert size were constructed using the SMRTbell Template Prep Kit v1.0, including DNA fragmentation, damage repair, blunt ligation, purification with 0.45× AMPure PB Beads and size selection with the BluePippin system. Library quality was assessed using the Qubit® 2.0 Fluorometer and Agilent 2100 Bioanalyzer. Sequencing was performed on the PacBio Sequel IIe platform (Pacific Biosciences of California, Menlo Park, CA, USA).

### Quality control and genome assembly

Short-read and long-read WGS data were trimmed using fastp (v0.23.2) and Filtlong, respectively. Contamination was assessed with Kraken2 (v2.1.2), excluding samples with <90% MTBC reads. Quality-filtered reads were processed using the bac-builder pipeline for hybrid assembly. The pipeline included basecalling and demultiplexing with Guppy, random subsampling with Rasusa and assembly with Flye, Canu, Raven and Miniasm/minimap2. A consensus assembly was generated with Trycycler and polished with Racon and Pilon. The genome start position was fixed to the *dnaA* gene using Circlator fixstart. The final quality of the assembled genomes was evaluated with QUAST and CheckM. In addition, BUSCO analysis was performed to determine genome completeness using the *mycobacteriales*_odb10 database as a reference.

### Genome annotation and variation detection

Consensus genomes were annotated using NCBI’s Prokaryotic Genome Annotation Pipeline (PGAP). Protein-coding genes were functionally annotated with InterProScan for KEGG pathways, Gene Ontology (GO) terms and Pfam domains. Whole-genome alignment to H37Rv_ref was performed using NucDiff (v2.0.3) to identify SNPs, insertions/deletions (InDels) and regions of difference, which were annotated with SNPEff.

For the 3,359 Lineage 2 strains included in this study, WGS analysis was performed uniformly by the authors using our in-house developed Snakemake-based pipeline, TBSeqPipe (https://github.com/KevinLYW366/TBSeqPipe). This pipeline ensures standardized and reproducible processing of Illumina paired-end sequencing data for MTBC isolates. Raw paired-end sequencing data were quality-filtered with fastp and screened for contamination with Kraken2. Reads were aligned to H37Rv_ref and A2018772 reference genomes using BWA-MEM. Samples with <90% genome coverage at ≥10× depth or average depth <100× were excluded. Variants were called using GATK4 (v4.2.6.1) with stringent criteria: ≥10× coverage, base quality ≥20, ≥5 supporting reads and ≥90% allele frequency for fixed SNPs. Variants were filtered to focus on high-confidence SNPs and InDels, excluding those in repetitive gene families (PE/PPE, PGRS), insertion sequences (IS*6110*) or drug resistance-associated regions (defined by the UVP pipeline, https://github.com/CPTR-ReSeqTB/UVP) using BEDtools (v2.30.0).

### Phylogenetic analysis

A concatenated alignment was generated from fixed SNPs, excluding those located in drug resistance-associated genes, polymorphic GC-rich PE (proline–glutamic acid) genes and other PE gene families. An approximately maximum-likelihood phylogenetic tree was then inferred from this alignment using FastTree. The tree was rooted with *Mycobacterium canettii* (RefSeq: NC_015848.1) as the outgroup and visualized on the Interactive Tree of Life (iTOL) website.

## Results

### Comparative genomic analysis of H37Rv_CN and H37Rv_ref

**Fig. 1.**
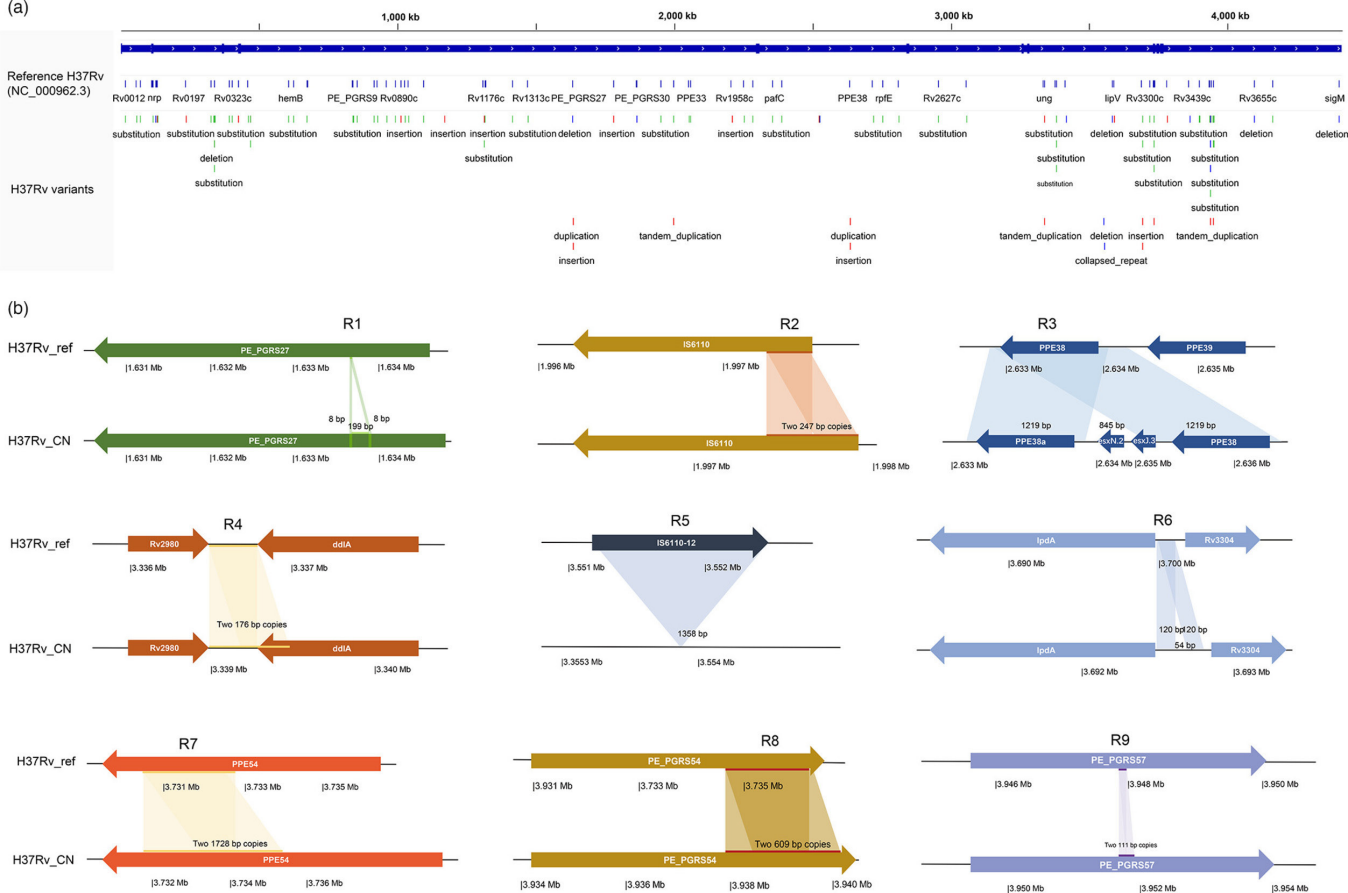
Comparative genomic analysis between H37Rv_CN and reference genome H37Rv. (a) IGV visualization of genome-wide variants in H37Rv_CN. (b) Characterization of nine SVs identified through DNAdiff analysis.

The genome of the locally preserved *M. tuberculosis* H37Rv strain from Zhejiang, China (H37Rv_CN), which was sequenced and *de novo* assembled by the authors in this study, was compared with the standard reference genome H37Rv_ref (NC_000962.3). The H37Rv_CN assembly resulted in a single circular chromosome of 4,415,456 bp (GC content 65.62%), encoding 3,954 protein-coding genes and 51 RNA genes. Quality metrics relative to H37Rv_ref showed 99.97% genome fraction, 0% contamination and 98.45% BUSCO completeness (Table 1, Data S1, available in the online Supplementary Material). Alignment with H37Rv_ref identified 145 SNPs, 49 insertions and deletions (InDels) and 14 structural variants (SVs) (Fig. 1a, Data S2). Among the SNPs, 84 caused nonsynonymous amino acid changes, potentially altering protein function.

**Table 1.** Basic genome information and quality control results for H37Rv_CN and A2018772

	H37Rv_CN	A2018772
**Assembly length**	4,415,456	4,410,965
**Number of contigs**	1	1
**N50**	4,415,456	4,410,965
**GC content (%**)	65.62	65.63
**Genome fraction (%**)	99.97	98.85
**Contamination (%**)	0	0
**BUSCO completeness (%**)	98.45	98.58
**Protein-coding genes**	3,954	3,888
**RNA genes**	51	51
**SNP count**	145	2,277
**InDel count**	49	373
**SV count**	14	219

Furthermore, 14 SVs were identified in 9 regions (R1–R9) in H37Rv_CN (Fig. 1b), including R1, a 207 bp in-frame insertion in PE_PGRS27 (*rv1450c*); R2, a 247 bp duplication of an IS*6110* transposase element; R3, a 2,064 bp replacement of PPE38 (*rv2351c*) with an intact upstream PPE38 copy, a truncated PPE38 paralog (PPE38a) and two novel *esx* gene paralogs (*esxN.2*, *esxJ.3*); R4, a 300 bp tandem duplication between *rv2980* and *ddlA*; R5, a 1,358 bp deletion spanning *rv3184* and *rv3185*; R6, a 120 bp duplication and 54 bp insertion in the *lpdA–rv3304* intergenic region; R7 and R8, 1,728 bp and 609 bp in-frame insertions in PPE54 (*rv3343c*) and PE_PGRS54 (*rv3508*), respectively; and R9, a 111 bp in-frame insertion in PE_PGRS57 (*rv3514*).

Functional annotation suggested that PE_PGRS insertions may contribute to antigenic variation and immune evasion [19]. The PPE38 replacement in R3 likely affects macrophage pro-inflammatory responses via the NF-κB pathway, given its role in regulating PPE protein expression [20]. The ddlA-associated duplication in R4 may influence peptidoglycan synthesis, a critical component of the cell wall [21]. Limited functional data are available for Rv3184, Rv3185, lpdA and other affected genes, necessitating further studies to elucidate their roles. These genomic differences highlight potential strain-specific adaptations in H37Rv_CN, which may impact downstream analyses of Lineage 2 strains when using H37Rv_ref as a reference.

### Genomic comparison of Beijing family strain A2018772 with H37Rv_ref

**Fig. 2.**
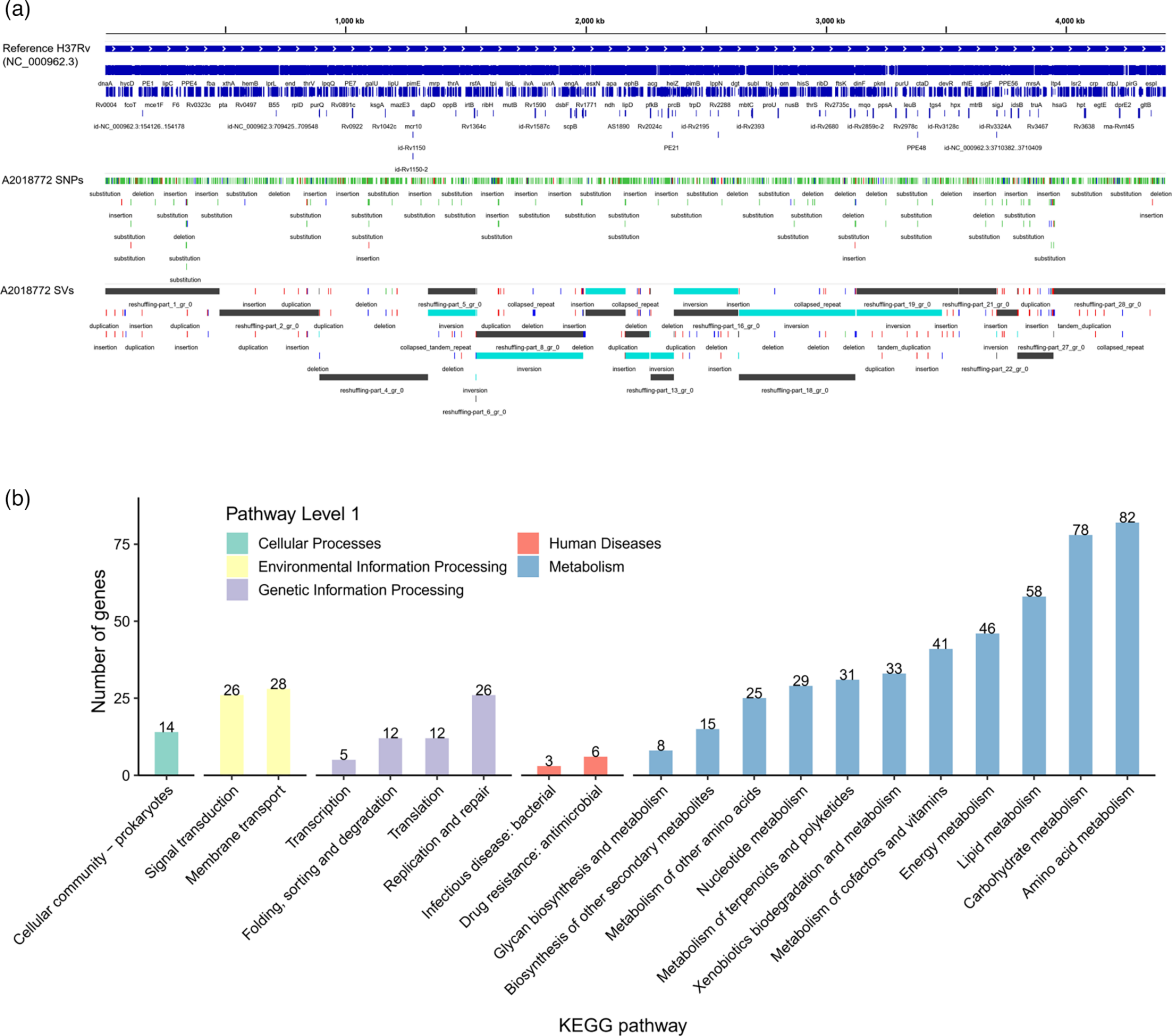
Comparative genomic analysis between Lineage 2 strain A2018772 and reference genome H37Rv. (**a**) Genome-wide mutation profile of A2018772 relative to H37Rv. (**b**) Frequency distribution of KEGG pathways classified in mutated genes of A2018772.

The complete genome of the Lineage 2 Beijing family strain A2018772, determined through WGS and *de novo* assembly in this study, was compared to the standard reference genome H37Rv_ref (NC_000962.3). The A2018772 assembly produced a single circular chromosome of 4,410,965 bp (GC content 65.63%), encoding 3888 protein-coding genes and 51 RNA genes. Benchmarking against H37Rv yielded a 98.85% genome fraction, 0% contamination and 98.58% BUSCO completeness (Table 1, Data S3). Alignment with H37Rv_ref identified 2,277 SNPs, 373 InDels, 219 SVs and 113 additional regions (Fig. 2a, Data S4). Functional annotation using KEGG pathways revealed these differences in five categories: cellular processes, human diseases, environmental information processing, metabolism and genetic information processing. The most pronounced differences were in metabolism, particularly amino acid, carbohydrate and lipid metabolism pathways (Fig. 2b), suggesting potential adaptations in A2018772’s metabolic capacity compared to H37Rv_ref (Data S5).

Among the numerous variants, we focused on nine with potential functional significance: one PPE deletion and eight SNPs. A 90 bp deletion in PPE8 (nucleotide 434,227, H37Rv_ref coordinates) removed 30 amino acids from the PE structural domain, potentially altering immune recognition [22]. Eight SNPs included a missense mutation (A→C) in *rv0988* (position 1,106,176) causing a tyrosine-to-serine substitution (Tyr354Ser); a missense mutation (G→A) in *mmsA* (*rv0753c*, position 845,542) resulting in an arginine-to-tryptophan substitution (Arg138Trp), potentially affecting dendritic cell activation and Th1 responses [23]; a missense mutation (C→T) in *rv1723* (position 1,950,068) leading to an arginine-to-cysteine substitution (Arg243Cys) in a putative hydrolase; a missense mutation (T→C) in *ubiA* (*rv3806c*, position 4,264,929) causing a leucine-to-tryptophan substitution (Leu177Trp), which may influence arabinogalactan synthesis and antibiotic susceptibility [24]; and a missense mutation (T→C) in *whiB6* (*rv3862c*, position 4,338,365) resulting in a cysteine-to-arginine substitution (Cys53Arg) in the Fe-S cluster core domain, likely disrupting regulatory function [25]. Additionally, three synonymous SNPs in *rv0197* (C→T, position 233,949), *rv2308* (G→T, position 2,580,877) and *rv2940c* (G→A, position 3,279,637) were identified, with no predicted functional impact. These variants highlight significant genomic divergence between Lineage 2 strain A2018772 and L4-based H37Rv_ref, potentially affecting virulence and transmission, which warrants further investigation in the context of lineage-specific genomic analyses.

### Prevalence of A2018772-derived SVs across public Lineage 2 Beijing family complete genomes

**Fig. 3.**
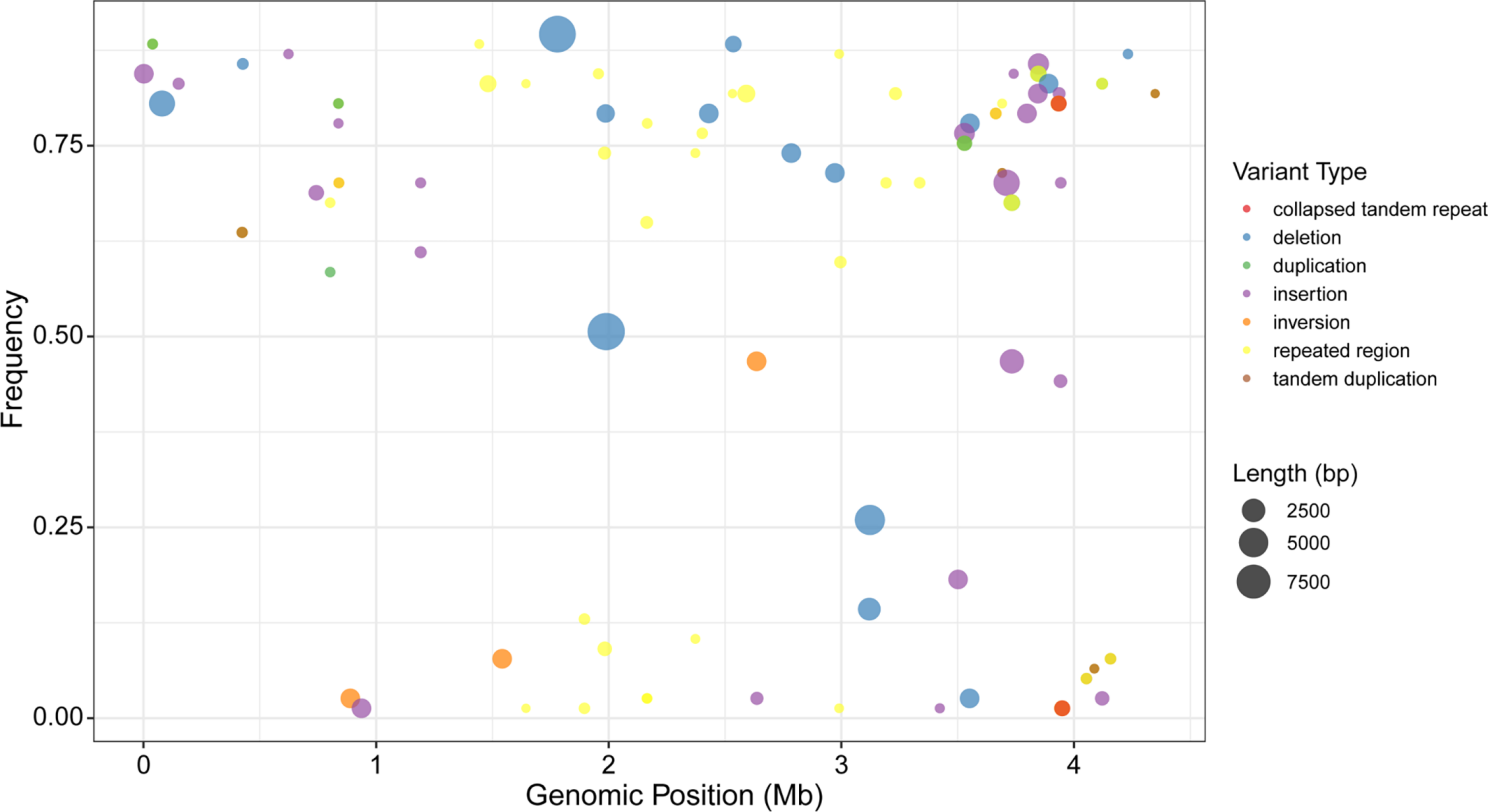
Genome-wide distribution of SVs and additional regions in Lineage 2 Beijing family strains. Each point represents a variant, with its position on the H37Rv reference genome (x-axis, Mb), population frequency (y-axis), functional type (colour) and length (size).

To validate whether the SVs and additional regions ≥50 bp identified in A2018772 are prevalent among Lineage 2 Beijing family strains, we analysed 76 publicly available complete genomes of Lineage 2 Beijing family strains (Data S6) by comparing each to the H37Rv reference. The results demonstrate that 117 large-scale mutations found in A2018772 were detected across the other 76 Lineage 2 Beijing family genomes (Data S7). Notably, multiple large deletions (>50 bp) occur at high frequencies – the largest being a 9,235 bp deletion (g.1,779,279–1,788,513) present in 92.2% (70/76) of the samples and a 710 bp deletion (g.2,535,432–2,536,141) found in 90.8% (69/76) of isolates. Similarly, large insertions and duplications are widespread, including a 1,763 bp insertion (at g.3,847,230) occurring in 88.2% (67/76) of samples and an 87 bp collapsed repeat (g.39,069–39,069) present in 90.8% (69/76) of the genomes (Fig. 3). Furthermore, genomic mapping confirms that these SVs are not clustered in specific regions but are dispersed throughout the chromosome, with only one high-density cluster observed ~3.5–4.0 Mbp, highlighting their broad genomic footprint.

### Lineage-specific reference (A2018772) improves resolution of transmission clustering in Lineage 2 Beijing family strains

**Fig. 4.**
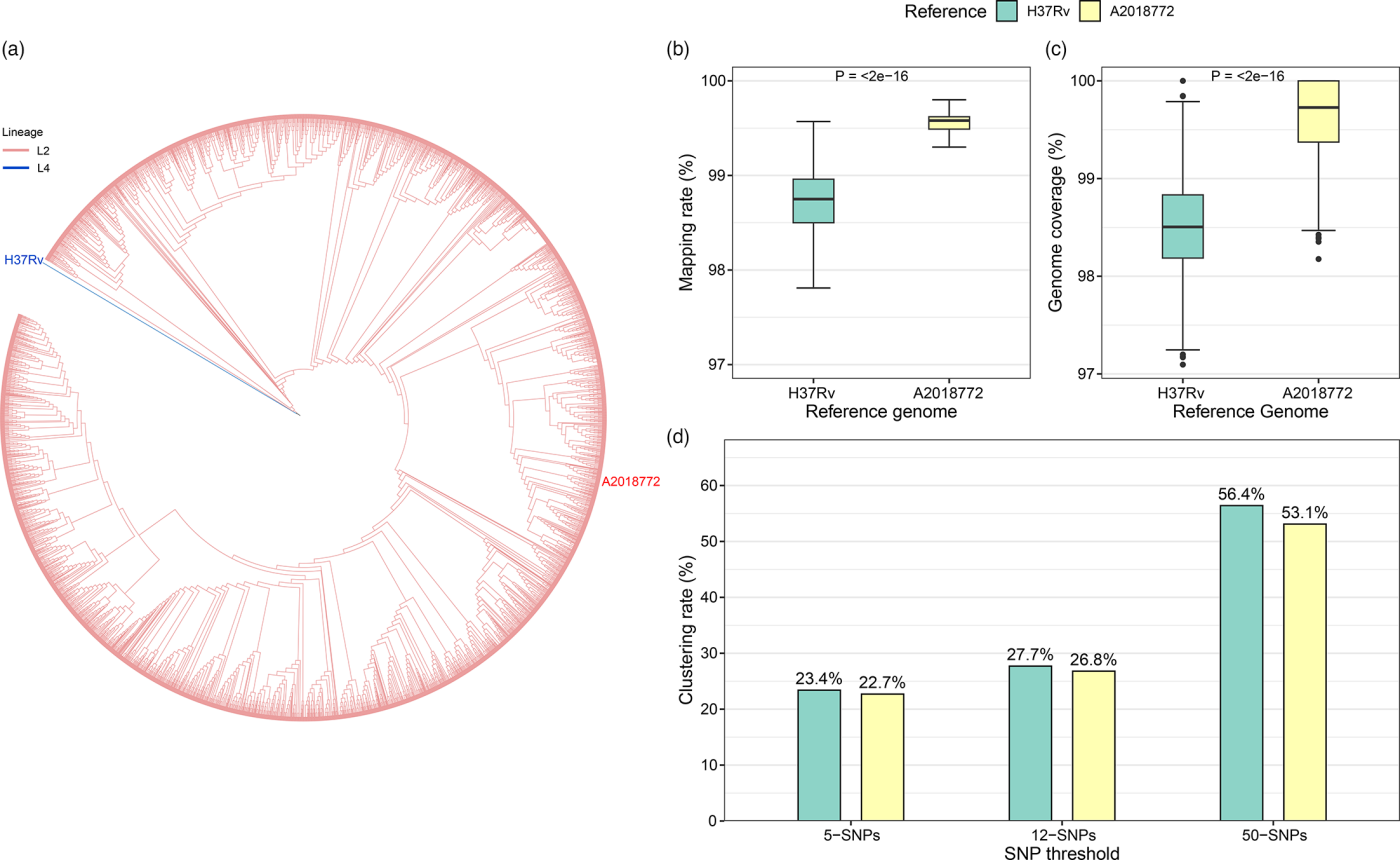
Comparative analysis of mapping performance and cluster resolution in Chinese Lineage 2 WGS data using A2018772 versus H37Rv reference genomes. (**a**) The phylogenetic tree includes reference genomes A2018772, H37Rv and the 3359 Lineage 2 strain from China. Branch colours indicate the respective lineages, while labels for H37Rv and A2018772 show their positions within the phylogenetic structure. (**b**) Boxplots of sequence alignment rates for both reference genomes. (**c**) Boxplots of sequencing coverage depth. Inter-group differences were statistically analysed using Wilcoxon signed-rank tests. (**d**) Bar plot comparing clustering rates between the two reference genomes at different SNP difference thresholds.

To assess the impact of reference genome selection on transmission cluster estimation, SNP detection was performed for 3,359 Lineage 2 strains (Data S8) isolated from China. Whole-genome alignments and variant calling for these strains were conducted by the authors using our standardized TBSeqPipe pipeline (see Methods for details), with consistent processing applied to both the H37Rv reference genome (NC_000962.3) and the A2018772 reference genome (Fig. 4a). Reads mapped to the A2018772 genome achieved a median alignment rate of 99.52% (Fig. 4b) and a median coverage of 99.73% (Fig. 4c), significantly higher than those obtained using the L4 H37Rv reference (98.72 and 98.50%, respectively), revealing that A2018772 better captures genetic information among conserved Lineage 2-specific genomic regions.

Using a 5-SNP threshold, 23.4% of strains (787/3,359) formed genomic clusters with at least 1 other isolate when mapped to H37Rv_ref, resulting in 285 clusters. In contrast, mapping to A2018772 produced fewer clusters (273, 22.7% clustering rate, 762/3,359) at the same threshold. Expanding the cluster difference threshold to 12 and 50 SNPs further revealed that the clustering rate using A2018772 as the reference remained lower than that obtained with H37Rv, and the difference between the two widened progressively. Specifically, at the 12-SNP threshold, the clustering rate for A2018772 was 26.8%, which was 0.9% lower than the rate for H37Rv (27.7%). At 50 SNPs, the gap increased to 3.3%, with A2018772 achieving a clustering rate of 53.1% compared to 56.4% for H37Rv (Fig. 4d). These differences suggest that using H37Rv_ref, an L4-based reference, may overestimate recent transmission events in Lineage 2 strains due to misalignment of lineage-specific variants. This finding highlights the importance of lineage-specific reference genomes for accurate transmission analysis in Lineage 2-prevalent regions.

## Discussion

This study provides compelling genomic evidence that challenges the conventional reliance on a single reference genome (H37Rv, RefSeq: NC_000962.3) for molecular epidemiological analyses of TB, particularly in regions dominated by Lineage 2 Beijing strains. By integrating hybrid sequencing (Illumina short-read and PacBio long-read technologies) [26], we resolved high-quality assemblies of the Chinese laboratory-adapted H37Rv (H37Rv_CN) and a clinical Beijing strain (A2018772), revealing substantial genomic divergences from the standard H37Rv reference (H37Rv_ref). These divergences – manifested as SVs, lineage-specific genes and metabolic pathway alterations – directly impact transmission clustering accuracy and necessitate a paradigm shift toward lineage-specific reference genomes for TB surveillance in endemic regions.

Our findings on H37Rv_CN genomic variations gain additional significance when contextualized with the recent comprehensive update to the H37Rv reference genome. We identified 145 SNPs, 49 InDels and 14 SV differences in H37Rv_CN compared to H37Rv_ref. Our analysis indicates that most discrepancies between H37Rv_ref and H37Rv_CN reflect technological limitations of the 1998 Sanger assembly rather than true genomic divergence. This conclusion is corroborated by the sequencing results of Poonam Chitale *et al*., who sequenced three H37Rv-1 strains using bact-bulder (an assembled sequencing pipeline) in 2022 with only 16 SNPs differing from ours (Data S9), validating the robustness of our hybrid approach. Among these SNPs, 12 are missense variants in coding regions (potentially altering protein function in genes such as fadD2, hemB, phoP and pstB, which are involved in fatty acid metabolism, haem biosynthesis, virulence regulation and phosphate transport, respectively), 3 are synonymous variants with likely minimal impact and 1 is in a non-coding splice region variant [27]. This 16-SNP variation could arise from microevolution during laboratory cultivation of the H37Rv strain in China. Given that ~10% of the *M. tuberculosis* genome consists of repetitive sequences, the short-read based Sanger Sequencing is likely to miss genuine elements, including pathogenicity-related genes such as esxN.2 and esxJ.3, potentially biassing studies of virulence and drug resistance [28]. Hybrid sequencing enables recovery of these missing regions and should be prioritized for updating the H37Rv reference, ideally from primary ATCC stocks to preserve its relevance as a global standard. Beyond technical artefacts, we detected true variations in H37Rv_CN, including insertions in IPdA and Rv2980, which likely arose during laboratory passage. Although *M. tuberculosis* is considered genetically conserved, WGS increasingly reveals within-host diversity and microevolution [29]. Bacteria accumulate mutations during passaging culture in the laboratory due to environmental factors such as laboratory pressure, media type, etc., which meets the principle of Darwinian natural selection. Although spontaneous mutation rates in bacteria are usually low, cumulative mutations resulting from certain mutations can dramatically increase the mutation rate; for example, the enterica serovar Typhimurium hypermutator strain had a 30-fold increase in point mutation rates compared with a wild-type strain [30]. These findings suggest that even a pathogen with low mutation rates can accumulate changes under selective pressures. MTB is generally recognized as a genetically homogeneous and relatively conserved bacterium, with a lower mutation rate than most bacteria and largely free of horizontal gene transfer events [31]. However, with the generalization of WGS technology, more complex TB infections have been discovered, and in addition to multiple infections, the presence of MTB strains with variable polymorphisms within the same host may also be due to microevolution of the same strain within the host. In Merker’s study, three MTB subclones were found to co-exist in a patient, showing a consistent VNTR pattern and a very low number of SNPs between them, presumably evolving in parallel during the last treatment [32]. However, so far, studies on the microevolution of MTB are relatively few and mostly based on the microenvironment in patients; there is no further evidence of significant adaptive mutations in *M. tuberculosis* during laboratory passaging cultures, which needs to be validated by further studies.

Lineage 2 and Lineage 4 significantly differ in virulence, immune response and transmissibility. Lineage 2 strains, particularly the Beijing lineage, are associated with higher virulence and transmissibility [33], often causing more severe disease presentations and inducing stronger innate immune responses in host macrophages [34]. L4 strains, while globally widespread, generally display lower virulence but maintain high transmissibility. Lineage 2 strains are predominant in East Asia, particularly in China, and are known for their ability to form large transmission clusters and accumulate drug resistance mutations. In contrast, L4 strains are prevalent in various regions worldwide and are linked to increased risks of disease progression and specific drug resistance patterns [35]. For the Beijing strain A2018772, the 2281 SNPs, 460 InDels and 219 SVs relative to H37Rv_ref highlight profound lineage-specific genomic architecture. Furthermore, research suggests that certain regions are specifically deleted in certain lineages, serving as effective markers for lineage identification [36,37]. Functional annotation revealed enrichment of variants in metabolic pathways – particularly amino acid, carbohydrate and lipid metabolism – suggesting Lineage 2-specific adaptations for nutrient acquisition or persistence in hostile host environments. Key mutations, such as the Arg138Trp substitution in mmsA (rv0753c), may impair dendritic cell activation and Th1 immunity [23], while the Leu177Trp change in ubiA (rv3806c) could perturb arabinogalactan biosynthesis, potentially altering cell wall integrity and antibiotic permeability [24]. What is more, the deletion in the PPE8 (Rv0355c) may give their potential to modulate Lineage 2-specific immune signatures; most members of this family are cell-wall-associated and play pivotal roles in inducing T-cell responses and suppressing macrophage apoptosis. They support MTB persistence by targeting host mitochondria to regulate immune signalling and programmed cell death pathways. These findings corroborate the hypothesis that Lineage 2 strains, through genomic streamlining and acquisition of novel genetic elements, have evolved distinct metabolic and virulence strategies compared to L4 strains. The widespread prevalence of Lineage 2-specific SVs (e.g. the 9.2 kb deletion in 89.7% of 78 public Beijing genomes) further validates A2018772 as a representative genomic scaffold for Lineage 2-dominated populations. Studying the expression function of its proteins at the genetic level can help to unravel this discrepancy, to find reasons for the widespread spread of Lineage 2 in recent years and the development of drug resistance and to search for new and reliable gene loci for treatment.

The most significant implication of this work lies in demonstrating how reference genome choice biases transmission inference. The reference genome serves as a fundamental benchmark in modern biological data analysis, providing the essential framework for a wide array of omics studies, including genomics, transcriptomics and metagenomics, and is crucial for enabling accurate and reliable downstream analyses [38]. As obtained from our sequencing results, there is a huge genetic difference between Lineage 2 and L4, with 2281 SNP differences. This difference will inevitably have implications for subsequent research. When applied to 3359 Chinese Lineage 2 strains, A2018772 as a reference yielded higher alignment rates (99.52% vs. 98.72%) and coverage (99.73% vs. 98.50%) than H37Rv_ref, reducing spurious SNP calls in lineage-specific regions like PE/PPE genes. Consequently, transmission clusters defined by a 5-SNP threshold decreased from 285 (H37Rv_ref) to 273 (A2018772), with the divergence widening at higher thresholds (e.g. 3.3% fewer clusters at 50 SNPs). This overestimation of clusters with H37Rv_ref likely stems from misalignment-induced artificial SNPs in Lineage 2-specific regions, inflating genetic distances and creating false transmission links. Similar biases have been found in Lineage 5, with potential overestimation of recent transmission when using H37Rv as the reference genome [39]. Our results thus provide empirical support for regionally tailored reference genomes to enhance the precision of molecular epidemiology. While another study found no necessity for employing lineage-specific reference genomes [40], their analysis was limited to L4 clinical strains and concentrated on SNPs and short insertions or deletions, neglecting larger gene deletions and SNPs within those regions. Conversely, our results demonstrate that aligning NGS data from Lineage 2 strains to an L4 reference genome has a significant effect on both the reference genome coverage and the coverage of lineage-specific genes. While the use of a single Lineage 2 genome as reference would have many benefits over H37Rv for particular study questions, several gene content differences were still observed within the Lineage 2. Indeed, insertions and deletions, as described for MTBC lineages, may be limited to sub-lineages. Research on pan-genomes has also opened new possibilities by integrating the entire Genbank of all MTBC lineages into a single pan-genome [41]. Studies have revealed small but significant differences in gene content between lineages, which often affect genes associated with virulence. While these differences are unlikely to impact drug resistance analysis, they may influence the delineation of transmission clusters. Given the close genetic relationships between MTBC strains and the absence of horizontal gene transfer events, constructing a pan-genome is feasible [42]. Nonetheless, this method remains underexplored, and it has certain limitations, including challenges in mapping reads spanning auxiliary genes and the boundaries of the core genome, etc.

From a public health perspective, this study directly addresses WHO End TB Strategy pillars – particularly ‘Integrated, Patient-Centered Care’ and ‘Bold Policies’ – by advocating for genomic tools optimized for local TB epidemiology. In China, where Lineage 2 strains drive >80% of TB cases, adopting A2018772 or similar lineage-specific references could refine resistance prediction (e.g. detecting Lineage 2-associated rpoBI491F mutations missed by H37Rv_ref), accelerate outbreak detection and optimize resource allocation. Moreover, the identified metabolic gene variations offer targets for novel diagnostics or therapeutics. For instance, inhibitors targeting Lineage 2-enriched lipid metabolism pathways could disrupt bacterial persistence in granulomas, where metabolic adaptations facilitate long-term survival.

## Limitations

This study has several limitations. One is the focus on a single strain (A2018772) for the identification of Lineage 2 differentiating regions; there are many subspectrums of Lineage 2 strains, and some of A2018772 mutations may be subspectrum-specific rather than common regions of the Lineage 2. Due to the small sample size, the results of the study may be subject to chance and may not be able to firmly determine whether the findings are generalizable to the broader population of strains. The other is a lack of functional validation. Studies have only identified some regions characteristic of Lineage 2 at the genomic level but have not validated the function of these regions. Genome-specific regions may contain genes or regulatory elements with important biological functions, or they may be non-functional sequences. Without functional validation, it is not possible to determine the specific impact of these endemic regions on the biological properties of the Lineage 2 spectrum (e.g. virulence, drug resistance, immune escape, etc.). Future studies should include a larger number of Lineage 2 strains to further characterize the genetic diversity and evolutionary history of this lineage. Additionally, functional studies are needed to validate the biological significance of the identified Lineage 2 differentiating regions.

## Conclusion

In this study, we performed WGS of a laboratory-preserved Chinese H37Rv strain and a pan-susceptible Lineage 2 strain. The novel H37Rv genome exhibited improved completeness compared with the 1998 reference, underscoring the need for updated assemblies to support accurate research. Several genomic regions unique to Lineage 2 were also identified, which may encode lineage-specific determinants of adaptation, virulence or drug resistance. Using these high-quality genomes, we conducted transmission clustering of 3,359 Lineage 2 isolates and found that analyses based on the Lineage 2 reference provided superior resolution compared with the standard H37Rv reference. These results emphasize three key points. First, the improved H37Rv genome offers a more reliable foundation for studies relying on this widely used reference, potentially revealing genetic elements overlooked in earlier versions. Second, the discovery of Lineage 2-specific genomic regions enhances understanding of lineage diversity within the MTBC and may inform future diagnostic, therapeutic and preventive strategies. Third, the use of lineage-appropriate reference genomes improves the accuracy of genomic epidemiology by better capturing within-lineage diversity and transmission dynamics, thereby strengthening public health applications. Collectively, our findings highlight the importance of refining reference genomes and incorporating lineage-specific resources into TB research. While H37Rv remains suitable for detecting resistance-associated mutations located in conserved core genes, lineage-specific references such as Lineage 2 provide critical advantages for evolutionary and epidemiological analyses.

## Abbreviations

DIN: DNA integrity number
gDNA: genomic DNA
LJ: Löwenstein–Jensen
MTB: *Mycobacterium tuberculosis*
MTBC: *Mycobacterium tuberculosis* complex
SV: structural variant
TB: tuberculosis
